# Comparative Assessment of Microbial Colonization and Tissue Reaction Among Three Suture Materials: A Randomized Controlled Trial

**DOI:** 10.3390/antibiotics14121265

**Published:** 2025-12-15

**Authors:** José Manuel Alarcón Cordovilla, María Victoria Olmedo-Gaya, María Teresa Arias-Moliz, Adela Baca García, David Sánchez-Porras, María Pilar Quesada-García, María Nuria Romero-Olid

**Affiliations:** 1 Independent Researcher, 18071 Granada, Spain; josemanu1998@gmail.com; 2 Department of Stomatology, School of Dentistry, University of Granada, Colegio Máximo s/n, 18071 Granada, Spain; mvolmedo@ugr.es (M.V.O.-G.); mpquesada@ugr.es (M.P.Q.-G.);; 3 Department of Microbiology, School of Dentistry, University of Granada, Colegio Máximo s/n, 18071 Granada, Spain; 4 Instituto de Investigación Biosanitaria ibs.GRANADA, 18012 Granada, Spain; davidsp@go.ugr.es; 5 Tissue Engineering Group, Department of Histology, School of Medicine, University of Granada, 18016 Granada, Spain

**Keywords:** inflammatory reaction, microbial colonization, oral surgery, sutures

## Abstract

**Background:** The aim of this study was to evaluate and compare the bacterial colonization, cytotoxicity, immune response, and clinical parameters of three different suture materials: multifilament silk (Silk^®^), monofilament nylon (Daclon^®^), and expanded polytetrafluoroethylene monofilament (PTFE^®^), in surgical extractions of impacted mandibular third molars. **Methods:** This randomized controlled clinical trial was conducted on twenty-one patients requiring surgical extraction of an impacted third mandibular molar. A bayonet-shaped flap was sutured using all three materials in each patient. Bacterial cell counting and qPCR were assessed for microbiological analysis. In vitro cytotoxicity was studied with the metabolic activity WST-1 assay. Inflammatory response was evaluated through histological analysis. Clinical parameters—healing, handling, slack, pain, swelling and trimus—were recorded. Statistical significance was set at *p* ≤ 0.05. **Results:** Monofilament sutures accumulated fewer bacteria and DNA copies than Silk^®^ (*p* < 0.05). The WST-1 assay revealed non-cytotoxic effects. Silk^®^ presented an immune response with lymphocyte-like cells. The highest values of pain and inflammation were reached at 48 h, with a significant correlation between them (*p* < 0.05). Silk and nylon were more manageable than PTFE (*p* < 0.001), and nylon had less slack (*p* < 0.001). **Conclusions:** Silk showed the poorest microbiological and histological performance, with higher levels of bacterial colonization and a more pronounced inflammatory response compared to the other types of suture. Clinically, it offered better handling than PTFE (PTFE^®^), comparable to nylon (Daclon^®^), but it exhibited greater slack, which could prove less favorable for wound stability. None of the sutures showed in vitro cytotoxicity. Monofilament sutures, particularly nylon (Daclon^®^), showed better outcomes, acceptable handling, less bacterial colonization, and a milder inflammatory response.

## 1. Introduction

Oral wound healing presents unique challenges due to a constant bacterial colonization of the soft tissues. This, combined with food debris and biofilm formation, increases the risk of surgical site infection (SSI). Additionally, the healing process is influenced by the continuous movement of oral tissues during functions such as speaking, swallowing, and chewing [1]. In surgical procedures requiring sutures, it is essential for clinicians to use materials well-suited to the oral environment.

Sutures can be classified based on their origin and stability in tissues as either natural or synthetic. Natural sutures degrade enzymatically, while synthetic ones undergo hydrolysis. Alternatively, sutures may be non-resorbable and they can be categorized as either monofilament or multifilament based on their macrostructure [2,3,4,5].

Sutures serve as new surfaces for bacterial adhesion and can contribute to inflammatory tissue reactions [3,4,5,6]. Monofilament sutures offer less resistance during passage through tissue and present a lower risk of microbial colonization compared to multifilament sutures. Although multifilament sutures are typically more resistant, their braided structure can facilitate through capillary action the transmission of oral fluids and therefore microorganisms into the wound—a phenomenon known as “wicking” [1,7].

Extraction of impacted third molars—the most common procedure in Oral and Maxillofacial Surgery—is associated with certain risks and postoperative complications that can affect patients’ quality of life [4,8,9,10]. Pain, typically most intense within the first 24 to 48 h, is reported as severe or intense in 12.3% of mandibular third molar extractions and 5.3% of maxillary third molar cases [11]. Other frequent complications include inflammation, trismus, and alveolar osteitis [4,6,8,10,12]. The incidence of postoperative infection varies between 0.8% and 5.2% [5,6,12,13,14], with infections tending to occur more frequently—around 5%—in lower third molars [12]. In these surgeries, sutures serve to reposition tissues and help prevent loss of the blood clot, thereby promoting healing [15]. Various strategies have been applied to reduce the morbidity following these procedures, which affects up to 79.6% of mandibular molar extractions [9,13].

Surgeons typically select suture materials based on factors such as the surgical site, expected time until suture removal, ease of handling, and tensile strength [14]. However, given the critical roles of inflammation and infection in postoperative healing, considerations of tissue reaction and bacterial adhesion are equally important. Despite this, few clinical studies in oral surgery have thoroughly examined bacterial colonization, inflammatory responses, or the impact of different suture materials on healing [1,14]. As postoperative outcomes largely depend on the performance and suitability of sutures, further research is needed [6]. The aim of the present study was, therefore, to evaluate and compare the bacterial colonization, in vitro cytotoxicity and in vivo inflammatory response, and clinical parameters of three different non-resorbable suture materials including multifilament silk, monofilament nylon and expanded polytetrafluoroethylene monofilament (PTFE), in surgical extractions of impacted mandibular third molars.

## 2. Results

**Figure 1:**
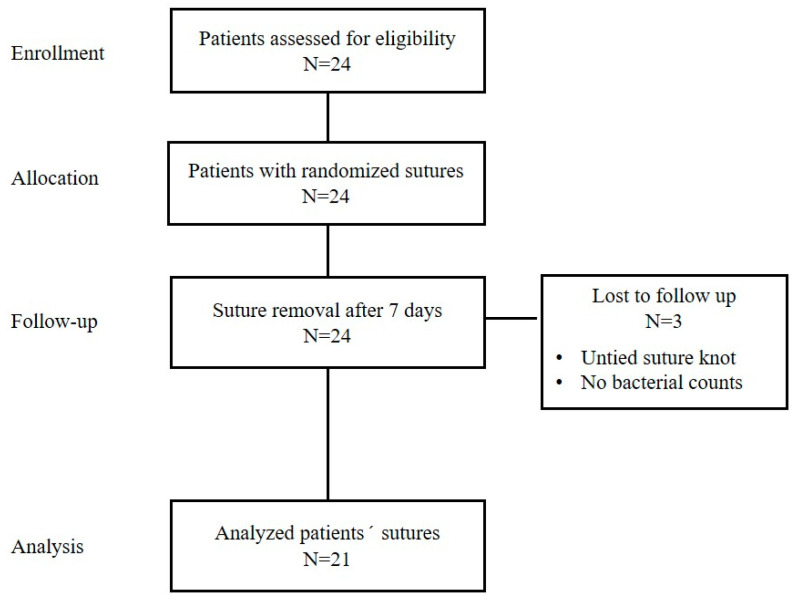
CONSORT flowchart of the study design phases.

A total of 24 patients were subjected to the surgical extraction of a lower third molar. Three patients were not included in the final analysis for different reasons: a knot was untied before its removal or absence of bacterial counts. Thus, the flow of 21 patients through the study is depicted in Figure 1.

The average age was 23 ± 3.54 years: 14 women (66.7%) and 7 men (33.3%). Most of the interventions were carried out on tooth 38 (71.4%), and 42.9% were very difficult according to the Pedersen scale (score 7–10). The main cause for extraction was pericoronitis during eruption, in 57.1% instances (Table 1).

### 2.1. Microbiological Analysis

Microbiological analysis gave significant differences among the three suture materials analyzed, in terms of CFU count (*p* < 0.001) as well as the qPCR assay (*p* < 0.001). For pairwise comparisons between sutures, Silk^®^ was found to be statistically different from PTFE^®^ and nylon (Daclon^®^, registering the highest bacterial values—with a mean of 6.15 CFU/mL and 10.11 of log DNA copies (Table 2). There were no significant differences between PTFE and nylon, although the latter showed slightly lower bacterial values than the PTFE. Blank and negative controls showed no amplification above background.

Assessment of potential PCR inhibition was performed indirectly through several quality controls. Extraction blanks showed no amplification with respect to the study samples; standard curves displayed consistent efficiency and linearity across runs; all samples and controls produced the expected melting-curve profiles and successful amplification.

### 2.2. Biocompatibility Analysis

#### 2.2.1. Direct Contact In Vitro Analysis

**Figure 2:**
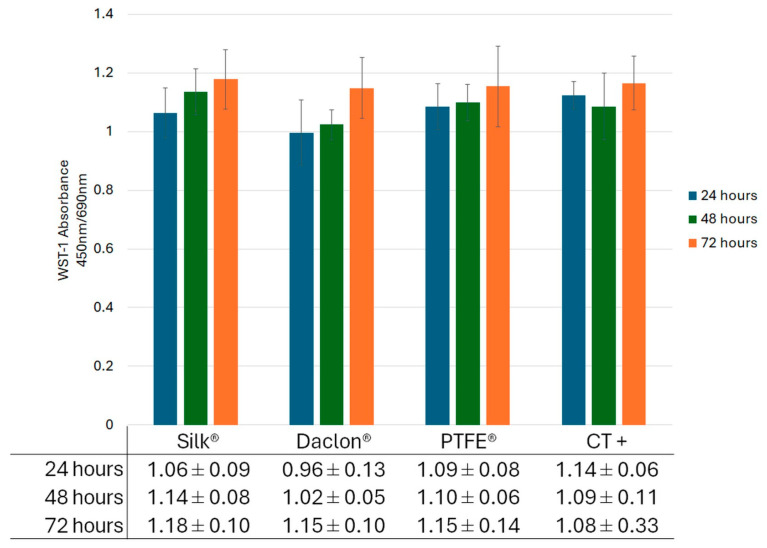
Quantitative absorbance results from the WST-1 (water soluble tetrazolium 1) assay performed at 24, 48 and 72 h during the direct contact in vitro biocompatibility test. Positive reaction was measured at 450 nm/690 nm. Cells cultured under standard conditions without sutures served as positive controls (CT+). Results are presented as mean ± SD. No significant differences (*p* > 0.05) were observed between groups at same point in time.

Cell metabolic activity results by WST-1 assay to determine in vitro biocompatibility revealed a time-increasing metabolic activity in all experimental conditions, with no significant differences between the different suture or CT+ groups at any time point of evaluation (*p* > 0.05) (Figure 2).

#### 2.2.2. Histological Analysis of Implanted Sutures

**Figure 3:**
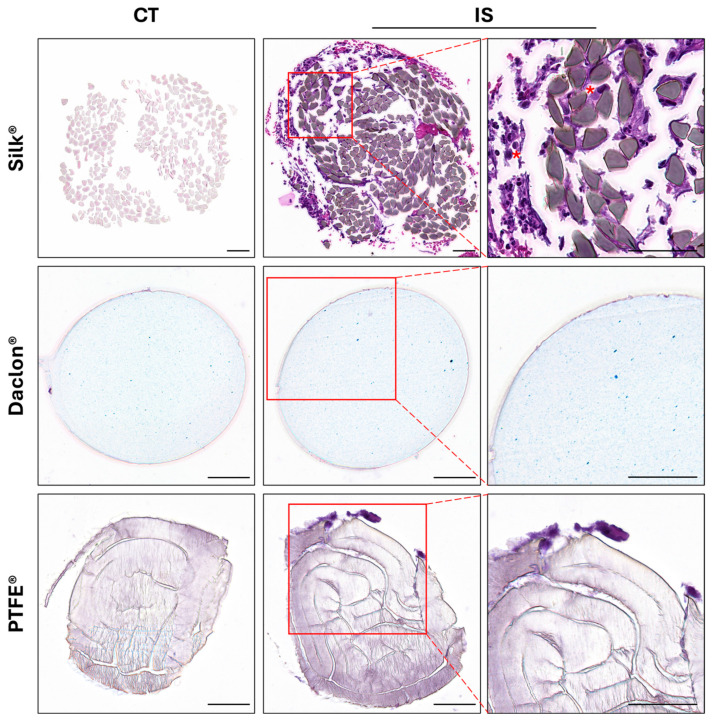
Representative hematoxilyn–eosine images of non-implanted control (CT) and implanted sutures (IS). A higher magnification of IS is shown to the right of the image, being observable lymphocytes and macrophages-like cells (red asterisk). Scale bar = 50 µm.

Histological evaluation by HE staining showed no significant structural differences between control and sutures after surgical implantation. However, the Silk^®^ group demonstrated a superior inflammatory response compared to other types of sutures, characterized by lymphocyte-like cells and stromal tissue adhering to the periphery of the suture, as well as cellular infiltration between the filaments. In contrast, nylon (Daclon^®^) sutures showed no signs of cellular infiltration or tissue adherence, while PTFE^®^ sutures exhibited minimal cell adhesion. Finally, no signs of filament degradation were observed in any of the suture groups during the evaluation period (Figure 3).

### 2.3. Clinical Parameters

With regard to pain and inflammation, the highest values recorded were at 48 h after the surgery, with mean scores of 4.05 ± 2.99 and 4.52 ± 3.46, respectively. A significant correlation was observed between the two variables (*p* < 0.05), indicating that patients experiencing higher levels of pain also tended to exhibit greater inflammation (Table 3). Trismus was absent in 11 patients (52.4%), yet among those who did (47.6%), in one case it was particularly severe, with a maximum mouth opening of only 16 mm (Table 4). Soft tissue healing after 7 days was generally favorable, with a mean healing score of 4.48 ± 1.54 on a 7-point scale. Approximately half of the patients (n = 12) required additional back-up medication. A statistically significant correlation was found between the level of pain reported on the day after surgery and the use of paracetamol (*p* = 0.03).

Regarding suture manageability, both Silk^®^ and Daclon^®^ received significantly better evaluations than PTFE^®^ (*p* < 0.001). Pairwise comparisons revealed no significant difference between Silk^®^ and Daclon^®^ (*p* > 0.05). However, in terms of slack, the nylon suture (Daclon^®^) showed the least slacking effect (*p* < 0.001), while no significant differences were observed between Silk^®^ and PTFE^®^ (*p* > 0.05) (Table 5).

## 3. Discussion

Suture material can significantly influence tissue healing and the risk of postoperative complications. In oral surgery, the ideal suture should be inert, non-allergenic, easy to handle, flexible, exhibit low memory, ensure knot stability and tensile strength, and cause minimal surgical trauma, bacterial contamination, and tissue inflammation [7,11,18,19,20]. The structural characteristics of the suture material play a crucial role in microbial colonization and the host’s inflammatory response [5,8,14,21,22], as does the possibility of coating sutures with certain antimicrobial materials such as triclosan [23], underscoring the importance of selecting an appropriate suture based on the specific procedure and surgical environment to minimize the risk of surgical site infections. Other relevant factors include the number and type of tissue layers involved, the depth of suture placement, wound tension, presence of edema, and the expected time until suture removal [11].

This clinical trial evaluated the performance of three types of suture materials. Silk remains the most widely used suture in oral surgery due to its key attributes, including physical strength, ease of handling, and low cost [1,11,15,18,24,25]. In contrast, PTFE and nylon are less commonly employed, though they offer distinct advantages. Both are monofilament sutures, consisting of a single continuous filament that reduces tissue friction, minimizes surgical trauma, and promotes healing [18]. PTFE is characterized by a smooth, soft surface and the absence of plastic memory, enhancing patient comfort and handling during placement [1,15,18,22]. Nylon, on the other hand, is known for its high tensile strength and excellent sliding properties [11,18,23]. Monofilament sutures such as PTFE and nylon may significantly reduce biofilm accumulation, which can positively affect the wound healing [26]. However, there is still a lack of studies directly evaluating bacterial colonization on monofilament sutures and its correlation with clinical parameters.

To ensure unbiased placement of the sutures, randomization was used to guarantee that each suture type was positioned equally across all sites, thereby eliminating potential confounding variables [1]. The study focused on the extraction of mandibular third molars, as these procedures are associated with a higher risk of infection due to limited surgical access, increased tissue trauma, and challenges in maintaining adequate oral hygiene in the area [9,10,12]. This surgical site harbors a high bacterial load, particularly in the pericoronal region, along with saliva stagnation—factors known to contribute to delayed healing [15]. Postoperative healing complications occur in approximately 22.6% of mandibular third molar extractions, compared to 13.3% for maxillary molars [27].

Sutures were removed after 7 days, aligning with the critical period for wound edge closure and initial tissue repair [11,12]. Postoperative antibiotics (specifically amoxicillin as no penicillin-allergic patients participated in the study) were prescribed for selected patients whose mandibular third molars were either fully or partially impacted and presented a surgical difficulty of 5 or more on the Pedersen scale—with 42.9% of cases scoring between 7 and 10. The administration of antibiotics does not appear to influence bacterial accumulation on the sutures [14,28] and seems to exert minimal and transient effects on the salivary microbiome [29]. All this highlights the limited effect of antibiotics on the microbiological analysis in the present study. In the microbiological study, two different assays were performed as there is no gold standard method to determine bacterial adhesion on a material’s surface [30]. Although the culturing technique allows for the recovery of viable bacteria, it fails to detect bacteria that lack the ability to grow in culture media, i.e., viable but nonculturable bacteria (VBNC) [31]. Since a high proportion of the bacteria present in the oral cavity pertain to this state, the culture method underestimates the total number of bacteria. Yet qPCR is a more sensitive technique and can detect microorganisms regardless of their growth phase [1,32].

Our results revealed significantly higher bacterial counts and DNA copy numbers in silk (Silk^®^) as compared to PTFE (PTFE^®^) and nylon (Daclon^®^), which showed similar profiles. These findings align with previous studies [1,2,4,5,7,8,15] that consistently report higher microbial accumulation on silk sutures than on PTFE. The braided structure of silk is thought to facilitate microbial adhesion [1,2,11,15,22], in contrast to the smooth surface of monofilament sutures like PTFE, which may hinder bacterial retention [14,33].

According to our histological analysis, a greater inflammatory reaction occurred with silk (Silk^®^), reflecting a clear immune response involving lymphocytic cells and stromal tissue adhered to the periphery, as well as cellular infiltration among the filaments. In contrast, the nylon samples (Daclon^®^ showed no infiltration or cellular adherence, and only a very scarce presence of cells adhered to the PTFE (PTFE^®^) sutures. These results are in line with previous findings, such as the study by Leknes et al. [34], who found that PTFE exhibited less adhesive properties than silk, both in the inflammatory tissue and inside the suture itself. In parallel, in order to determine whether the histological results could be influenced by the chemical composition of the material, the WST-1 test indicated an increase in metabolic activity over time, without significant differences between the control and the different sutures. This confirms that the chemical composition of the sutures does not affect cell viability, i.e., they would most likely not release harmful substances inducing cell damage once implanted. Moreover, previous studies show that the capacity of a suture material to produce infection is related to the profile of capillary absorption and the suture fluids, monofilaments having a lower friction coefficient, thus producing less tissue damage and bacterial accumulation [1,35]. Our histological and cytotoxicity results confirm this hypothesis, showing a greater inflammatory reaction, but not cytotoxicity, in the multifilament experimental group of the silk (Silk^®^), when compared to the monofilament sutures of nylon (Daclon^®^ and PTFE^®^) [2,25].

One limitation of this study is the inability to assess in situ degradation of the sutures. The 7-day observation period was insufficient to detect potential filament degradation through histological analysis. Nonetheless, despite being classified as non-resorbable, our findings suggest that the silk suture (Silk^®^) may degrade earlier than other materials due to the greater cellular infiltration observed. This could be attributed to its composition, which includes approximately 10% water, potentially making it more susceptible to degradation under oral conditions [1].

Clinical features showed a peak of pain and inflammation 48 h after surgery, as well as a positive association between the two parameters. The association of greater postoperatory inflammation with multifilament configuration (as opposed to monofilament) has been previously reported [5,15,18] and may be influenced by a greater accumulation of bacteria [4,5,8,10,11]. Half of the patients in our study had to take back-up analgesic medication, and a positive correlation was seen between pain on the second day and the intake of paracetamol. Healing was favorable at 7 days.

Proper closure of the extraction site is essential for optimal tissue healing. Loose sutures result in less close contact between the thread and the underlying tissue, potentially creating spaces that hinder epithelial regeneration. Still, overly tight closure should also be avoided, as it could cause localized ischemia and increase the risk of infection [36]. In our study, nylon (Daclon^®^) demonstrated significantly less slack compared to silk (Silk^®^) and PTFE (PTFE^®^), while silk and PTFE performed similarly, with no statistically significant differences. These findings are consistent with previous reports [1,2,18], suggesting that monofilament sutures such as nylon and PTFE exhibit less postoperative slack than braided silk, thereby enhancing wound stability and reducing the likelihood of dehiscence. Moreover, PTFE has shown superior dimensional stability, maintaining its tensile strength even after 21 days of immersion in artificial saliva [33].

In terms of suture manageability, silk (Silk^®^) and nylon (Daclon^®^) performed better than PTFE (PTFE^®^). Silk is often favored by clinicians due to its ease of handling [2], although some authors [11] have noted that nylon also offers excellent manageability—even in very fine calibers—along with good tensile strength and elasticity. Regarding knot security, monofilament sutures are generally more prone to untying [11]; and based on our experience, they can also cause discomfort for patients, particularly in the case of nylon.

The selection of suture material, along with rigorous postoperative care, plays a critical role in promoting wound healing and minimizing surgical complications, especially in the extraction of lower third molars, associated with higher postoperative morbidity. The best overall outcomes were observed with the monofilament sutures, particularly nylon (Daclon^®^), which demonstrated superior handling, lower bacterial colonization, a milder inflammatory response, and reduced slack compared to PTFE (PTFE^®^). However, the final choice of suture should also consider the clinician’s preferences and the specific requirements of each surgical case.

## 4. Materials and Methods

### 4.1. Registration and Ethical Approval

The study was approved by the Ethics Committee (reference no. 2028/CEIH/2021) and registered on ClinicalTrials.gov (NCT06864559). The clinical trial was conducted in accordance with the CONSORT 2025 guidelines [37]. All participants provided written informed consent in accordance with the Declaration of Helsinki.

### 4.2. Study Design and Patient Selection

This randomized controlled clinical trial was conducted among patients undergoing extraction of mandibular third molars, recruited through the Master’s Degree Program in Oral Surgery and Implantology at the School of Dentistry of the University where the study was performed. Inclusion criteria were: patients classified as ASA I according to the American Society of Anesthesiology, non-smokers, aged 18 or older, and requiring surgical extraction of a mandibular third molar—either totally or partially impacted—with a difficulty score of 5 or higher on the Pedersen scale [16,17]. Exclusion criteria included pregnancy or nursing, ASA classification II to V, antibiotic use within one week prior to surgery, and hypersensitivity or allergy to suture material.

The sample size was estimated to be adequate according to Wittes [38]. The sample size calculation was performed using G*Power 3.1.9.4 based on data from previously published studies [9,11]. The design with 20 subjects allows reaching a power of 81% to detect a medium effect size of 0.30, with α = 0.05 and a power of 0.80 (1 − β error probability). The total number was increased to 24 patients to compensate for possible losses.

### 4.3. Suture Materials

Three types of suture materials were used in this study:

Natural, non-resorbable, braided silk (Silk^®^, Laboratorios Normon, Madrid, Spain)

Synthetic, non-resorbable, monofilament nylon (Daclon^®^, Sanhigia, Zaragoza, Spain)

Synthetic, non-resorbable, monofilament expanded polytetrafluoroethylene (e-PTFE^®^; Sanhigia, Zaragoza, Spain).

All sutures used were 4-0 reverse cutting needles with a 3/8 circle curvature.

### 4.4. Surgical Protocol

The surgical procedures were carried out by two oral surgeons (M.N.R.O., M.V.O.G.). One week before the tooth extraction the patients received oral hygiene instructions, followed by professional mechanical plaque removal (PMPR) if deemed clinically appropriate. Immediately after surgery, patients rinsed for 30 s with 0.12% chlorhexidine mouthwash (Perio-Aid^®^; Dentaid, Barcelona, Spain). Their lips and perioral skin were disinfected using 0.2% chlorhexidine digluconate (Corsodyl; Haleon, Brentford, UK) applied with sterile applicators. Local anesthesia was administered using 4% articaine with 1:100,000 epinephrine (Ultracain^®^; Normon, Madrid, Spain).

A full thickness incision was made to expose the third molar and adjacent bone using a bayonet flap design. Osteotomy was conducted using a straight hand-piece and round bur, sectioning the tooth when necessary with a high speed bur. Primary closure was achieved through simple interrupted sutures, using all three materials studied, placed randomly with the aid of a web-based tool (https://www.sealedenvelope.com/simple-randomiser/v1/lists; URL accessed the 11 December 2025) and the allocation was assigned by M.T.A.M. The sutures were placed in the three different positions of the incision following the computer-generated randomized numbers. In this way, two sutures of the same material were in the horizontal crestal incision (position 1), another suture in the superior portion of the releasing incision (position 2), and the third and final one in the inferior portion of the releasing incision (position 3). Each suture material was therefore placed in each position the same number of times (total, 8 times). Only one suture in position 1 was used for the study and the other was discarded.

The patients were given post-operative instructions that included the use of systemic antibiotics (5 days of 8-hourly 750 mg of amoxicillin, or 300 mg of clindamycin 300 for patients allergic to penicillin), 0.12% chlorhexidine oral rinses with Perio-Aid^®^ twice a day, and application of 0.20% chlorhexidine +0.20% hyaluronic acid gel (Perio-Aid^®^) three times per day. For pain control, ibuprofen 400 mg was prescribed, every 8 h for the first two days post-op; as back-up analgesic medication, paracetamol was prescribed: 1 gr every 8 h in the event of persistent pain, to be taken one hour after the ibuprofen.

The sutures were removed 7 days post-op. Each suture was sectioned into two parts, four millimeters corresponding to the part outside the wound taken for the microbiological study, and the part that remained inside the wound was introduced in tubes with formaldehyde for the histological study [1].

### 4.5. Microbiological Analysis

#### 4.5.1. Culture Assay

For the microbiological study, each 4 mm suture section was introduced in an Eppendorf tube with 500 µL of BHI broth (Brain Heart Infusion, Scharlau Chemie S.A., Barcelona, Spain). The bacteria adhered to the different sutures was resuspended in the BHI medium after shaking in vortex for 10 s, followed by 10 min in ultrasound (J.P. Selecta S.A., Barcelona, Spain).

For the culture assay, once the bacteria were recovered, 5 dilutions were made (10-1-10-5) and 10 µL of each dilution was streaked on blood agar plates (Maim S.L., Zaragoza, Spain), which were then incubated in anaerobic conditions at 37 °C during 7 days. The colony-forming units per milliliter (CFU/mL) were counted.

#### 4.5.2. qPCR Assay

The DNA of the samples in BHI broth was extracted using the QIAamp DNA Mini Kit (Qiagen, Valencia, CA, USA), following the protocol recommended by the manufacturer and preceded by a 30 min incubation with 10 mg/mL lysozyme in tris-chloride buffer at 37 °C. Blank controls with no sample were included in the extraction process. To quantify the total bacterial load, 16S rRNA gene-targeted qPCR was performed using the QuantStudio 6 High-throughput Realtime PCR system (Thermo Fisher Scientific, Waltham, MA, USA) [39]. SsoFast EvaGreen Supermix (Bio-Rad Laboratories, Hercules, CA, USA) was used for the master mix in a total reaction mixture volume of 10 μL. The universal bacterial primers were F: 5′-AAACTCAAAKGAATTGACGGGG-3′ and R: 5′-GGGTTGCGCTCGTTRYGG-3′. Primers in a final concentration of 0.5 μM each and DNA extract volume of 1 μL were added to the PCR master mix, in MicroAmp Fast 96-Well reaction plates. Cycling conditions included 95 °C for 10 min and 40 cycles consisting of denaturalization at 95 °C for 15 s and annealing at 60 °C for 1 min. Negative controls containing no template DNA were subjected to the same procedures. The specificity of the amplified products was determined by analysis of melting curves, which were obtained using QuantStudio(TM) 6 Flex System default parameters. Fluorescence thresholds were automatically determined by the software for each run, and Ct values were defined as the cycle at which fluorescence crossed this threshold. The number of DNA copies per sample was obtained based on standard curves, for which known concentrations of *Escherichia coli* 16S gene were used. All samples, extraction blank, negative controls and the standard curve were analyzed in duplicate.

### 4.6. Biocompatibility Analysis

#### 4.6.1. Direct Contact In Vitro Analysis

To perform the in vitro biocompatibility test, human fibroblast derived from oral mucosa (HFOM) obtained from healthy patients under informed consent were subjected to direct-contact culture with the different suture types.

Tissue samples were washed in phosphate-buffered solution (PBS) supplemented with 2% antibiotic-antimycotic solution (Merck, Darmstadt, Germany). The samples were digested overnight in a collagenase type I solution (Gibco, Alcobendas, Madrid, Spain), and centrifuged; cell pellets were cultured with DMEM supplemented with 10% fetal bovine serum (FBS) and 1% antibiotic-antimycotic solution (Merck) in standard culture conditions (37 °C and 5% CO_2_).

Then, 3 × 10^3^ HFOM of passage II per well were seeded in a 96-wells plate. After 24 h, the culture medium was replaced with 100 μL of fresh medium and a 3 mm fragment of each suture type (Silk^®^, Daclon^®^ and PTFE^®^) was added, in 6 independent wells per experimental group and time of evaluation (n = 6). After 24, 48 and 72 h of direct contact, the cell metabolic activity water soluble tetrazolium 1 (WST-1) (Merck) assay was performed: incubating a 10% WST-1 working solution diluted in culture medium for 2 h. The colorimetric reaction was measured at 450 nm/690 nm with an ASYS UVM340 spectrophotometer using the software DigiRead v1.5 (Biochrom Ltd., Cambridge, UK). Positive (CT+) and negative (CT−) controls were run under the same experimental conditions but without sutures. For CT−, cells were treated with 2% TRITON X-100 solution in PBS to induce irreversible cell damage. All values were normalized with the 10% WST-1 working solution incubated without cells.

#### 4.6.2. Histological Analysis of Implanted Sutures

After 7 days since implantation, the extracted sutures and non-implanted controls were fixed for 48 h in 3.7–4% neutral buffered formaldehyde, dehydrated in increasing ethanol solution and embedded in paraffin (Panreac, Barcelona, Spain). Sections of 5 µm were obtained, then hematoxylin-eosin (HE) (Kalamazoo, MI, USA) stained following standard procedures. Histological images were obtained with a Nikon Eclipse Ti-U direct microscope (Nikon, Tokyo, Japan). The histological evaluation was performed by two members of the Department of Histology of the University of Granada (D.S.P. and another researcher).

### 4.7. Clinical Parameters

Both the pain and inflammation of the patient were assessed at 24 h, 48 h and 7 days after suture removal by means of the visual analogical scale (VAS) (0 = no pain/no inflammation, to 10 = worst pain/maximum inflammation). The trimus or limitation of the mouth opening was measured (in mm) as the distance between the incisal edges of the upper and lower central incisors previous to the surgery and at seven days thereafter. Also at 7 days the degree of soft tissue healing was evaluated by means of the Pippi index [40], which takes into account the redness of granulation tissue, bleeding, suppuration, swelling, pain on palpation, degree of tissue epithelialization and pain, with a total score of 7 for better healing. Noted as well was the need for back-up analgesia with 1 g of paracetamol (no/yes).

Finally, the handling and slack of the different suture materials was also evaluated. Intra-operatory ease in handling when suturing was appraised through the VAS. Slack was measured twice per site by a single calibrated operator, and the mean of the two measurements was used for analysis. Measurements were performed using a standardized technique and a periodontal probe calibrated in such a manner that the knot could be carefully raised with cotton pliers, and the distance from the knot to the soft tissue could be measured with a precision of 0.5 mm.

### 4.8. Statistical Analysis

For analysis of the variables CFU/mL and DNA copies, the decimal logarithmic transformation was used (log10 + 1 when a value was equal to 0). The Shapiro–Wilk test was applied to determine if the variables of study followed a Gaussian distribution, showing that they were not normal. The comparative analysis of the different types of suture relied on the test of Friedman, while the pairwise comparisons were made with the Wilcoxon test. For analysis of bivariate correlations, the Spearman correlation coefficient was used, calculating the coefficient of linear determination R^2^ and analyzing its significance. The level of significance was set at *p* < 0.05. Statistical analysis was performed with the program IBM SPSS Statistics (v.29).

## 5. Conclusions

Silk (Silk ^®^) showed the poorest microbiological and histological performance, with higher levels of bacterial colonization and a more pronounced inflammatory response compared to the other types of suture. Clinically, it offered better handling than PTFE (PTFE^®^), beingcomparable to nylon (Daclon^®^), but it exhibited greater slack, which could be less favorable for wound stability. None of the sutures showed in vitro cytotoxicity. Monofilament sutures, particularly nylon (Daclon^®^), showed better outcomes, acceptable handling, less bacterial colonization, and a milder inflammatory response.

## Figures and Tables

**Table 1:** Description of the clinical and demographic variables of the patients at baseline (*n* = 21 in all variables).

Variable		No. of Patients (%)
Demographics	Gender (male/female)	7/14
	Age ^1^ (years)	23 ± 3.54 (19–33)
Localization	Lower third molar (right/left)	6/15
Position		
	Mesio-angular	5 (23.8)
	Horizontal	2 (9.5)
	Vertical	7 (33.3)
	Disto-angular	7 (33.3)
Depth		
	Level A	2 (9.5)
	Level B	13 (61.9)
	Level C	6 (28.6)
Branch mandibular relation		
	Class 1	5 (23.8)
	Class 2	9 (42.9)
	Class 3	7 (33.3)
Difficulty ^2^		
	Moderate	12 (57.1)
	Difficult	9 (42.9)
Indication for surgery		
	Associated pathology	12 (57.1)
	Preventive extraction	7 (33.3)
	Orthodontics	1 (4.8)
	Second molar injury	1 (4.8)

^1^ Mean ± standard deviation (range). ^2^ Pedersen scale [16,17].

**Table 2:** Comparison of Log_10_ colony-forming units (CFUs) and Log_10_ DNA copy numbers among different suture types. Mean ± standard deviation (median; minimum–maximum).

	Log_10_ CFUs	Log_10_ DNA Copies
Silk^®^	6.15 ± 1.34 (6.43; 2.30–7.94) ^a^	10.11 ± 1.27 (10.52; 6.34–12.14) ^a^
Daclon^®^	3.75 ± 2.14 (4.53; 0.00–6.54) ^b^	8.56 ± 1.46 (9.15; 5.37–10.48) ^b^
PTFE^®^	3.87 ± 2.11 (4.48; 0.00–6.62) ^b^	8.66 ± 1.28 (9.20; 5.50–10.09) ^b^
Comparison *p*-value *	<0.001	<0.001

* Global comparisons by Friedman test (previously Shapiro–Wilk test determined that the variables do not follow a normal distribution). Read vertically, the same superscript letter shows no significant differences by Wilcoxon test.

**Table 3:** Analysis of the association between pain and inflammation during the evolution of their scores over time (n = 21 patients). Mean ± standard deviation (minimum–maximum).

	Day 1	Day 2	Day 7	Comparison *p*-Value ^1^
Pain	2.57 ± 2.62 (0–8) ^a^	4.05 ± 2.99 (1–10) ^a^	0.67 ± 1.02 (0–3) ^b^	<0.000
Inflammation	2.19 ± 2.32 (0–7) ^a^	4.52 ± 3.46 (0–10) ^b^	0.38 ± 0.74 (0–2) ^c^	<0.000
Correlation ^2^	0.26 (*p* = 0.25)	0.44 (*p* < 0.05) *	0.35 (*p* = 0.12)	

^1^ Global comparison by Friedman test. Read horizontally, the same superscript letter shows no significant differences by Wilcoxon test. ^2^ Spearman correlation coefficient: R (*p*-value). * Statistically significant *p* < 0.05.

**Table 4:** Maximum mouth opening at baseline and seven days after surgery (n = 21).

Maximum Mouth Opening	Mean (SD)	Minimum	Maximum
Baseline	46.81 (4.60)	40	57
Day 7	44.95 (4.71)	35	55
Comparison *p*-value	0.004 *		

* Statistically significant, Wilcoxon test *p* < 0.05. SD: Standard deviation.

**Table 5:** Comparison of handling and slack results among different suture types. Mean ± standard deviation (median; minimum–maximum).

	Handling ^1^	Slack ^2^
Silk^®^	7.67 ± 1.35 (8; 5–9) ^a^	2 ± 1 (2; 0–4) ^a^
Daclon^®^	7.33 ± 0.86 (7; 6–9) ^a^	0.38 ± 0.50 (0; 0–1) ^b^
PTFE^®^	5.90 ± 1.61 (6; 2–9) ^b^	1.57 ± 1.03 (1; 0–4) ^a^
Comparison *p*-value *	<0.001	<0.001

* Global comparison by Friedman test (previously Shapiro–Wilk test determined that the variables do not follow a normal distribution). Read vertically, the same superscript letter shows no significant differences by Wilcoxon test. ^1^ Handling measures use Visual Analogue Scale. ^2^ Slack is measured in millimeters.

## Data Availability

The data presented in this study are available on request from the corresponding author.
